# Establishment of Biomimetic Soft Tissue Integration with the Surface of Zirconia Fused with Platelet-Activating Peptide

**DOI:** 10.3390/ma15134597

**Published:** 2022-06-30

**Authors:** Chia-Yu Chen, Wonwoo Jang, David M. Kim, Masazumi Nagai, Shigemi Nagai

**Affiliations:** Department of Oral Medicine, Immunity and Infection, Harvard School of Dental Medicine, 188 Longwood Avenue, Boston, MA 02115, USA; won8450@gmail.com (W.J.); dkim@hsdm.harvard.edu (D.M.K.); masazumi_nagai@hms.harvard.edu (M.N.); shigemi_nagai@hsdm.harvard.edu (S.N.)

**Keywords:** implant surface, zirconia, epithelial attachment, soft tissue sealing

## Abstract

Soft tissue sealing around zirconia (ZrO_2_) abutment is critical for the long-term stability of dental implants. The goal of the study is to develop a strong basal lamina (BL)-mediated epithelial attachment to ZrO_2_ via a novel physicochemical immobilization method. An electrophoretic fusion (EPF) method was applied to fuse a phosphonic acid (PA) linker to ZrO_2_ discs. Bindings of the PA linker and the following protease activated receptor 4 (PAR4) were verified by Fourier-transform infrared spectroscopy (FITR). Then, ZrO_2_ discs were doped in platelet-rich plasma (PRP). Platelet-derived growth factor (PDGF) was measured to assess platelet activation. PRP-doped discs were subsequently co-cultured with human gingival epithelial cells (OBA9) to evaluate establishment of basal lamina-mediated epithelial attachment. The EPF method achieved robust immobilization of the PA linker and PAR4 onto the ZrO_2_ surface. The resultant PAR4-coupled ZrO_2_ successfully induced platelet aggregation and activation. Furthermore, a BL-mediated epithelial attachment was established. The results are significant for clinical application to minimize the risk of developing peri-implant diseases.

## 1. Introduction

Modern implant therapy yields excellent long-term results, as documented by numerous 10-year studies with success and survival rates higher than 95% [1,2]. However, research has shown that after 5 to 10 years, peri-implantitis occurs in between 12% and 66% of patients and peri-implant mucositis occurs in about 80% [3,4,5]. Peri-implant mucositis is defined as an inflammatory lesion of the soft tissues surrounding an endosseous implant in the absence of the loss of supporting bone. Without effective treatment, mucositis eventually advances to peri-implantitis, a pathological condition characterized by inflammation in the peri-implant connective tissue and progressive loss of supporting bone.

The peri-implant mucosa is consistently exposed to microorganisms in the biofilm present on implant surfaces [6] and is more prone to biofilm invasion leading to bone loss compared to the natural dentition due to the lack of basal lamina (BL)-mediated epithelial attachment. The establishment and maintenance of healthy soft tissues around implant abutments are important for the long-term service of the implant [7].

Numerous clinical and experimental studies have shown that the critical difference in the epithelial attachment between a natural tooth and the dental implant is BL-mediated tight sealing. The increased number of cases of peri-implant mucositis/peri-implantitis can be attributed to the lack of BL-mediated sealing. Junctional epithelium (JE) is a specialized gingival epithelium located at the junction of periodontal soft tissue and hard tissue that attaches to the crown or root similar to a collar via hemidesmosomes in internal basal lamina (IBL) [8]. This epithelial attachment not only provides the primary barrier to microbial entry but also allows physiologic defense by penetrations of polymorphonuclear cells (PMNs) and lymphocytes within JE [9,10]. The peri-implant epithelium (PIE) is crucial to the seal between the oral environment and the implant surface. However, the PIE is mechanically more fragile, with a lack of tight sealing compared to the natural teeth epithelium due to the inferior quality and quantity of JE attachment [11,12]. When subjected to bacterial challenge, the destruction of PIE is faster and more devastating than in periodontal tissue of natural dentition [13,14]. Therefore, enhancement of the implant epithelial PIE sealing will increase implant survival [7].

Several studies have investigated the effect of implant surface modification with or without biological agents on epithelial cell and fibroblast attachment to titanium surfaces. Although those studies showed enhanced gingival fibroblast and epithelial cell attachment, proof of essential epithelial attachment (hemidesmosome contact on the titanium surface) was lacking [15,16,17]. In our previous study, titanium (Ti) surface modification with a protease activated receptor 4-activating peptide (PAR4) achieved BL-mediated epithelial attachment via platelet aggregation and activation [18]. PAR4 mimics thrombin-mediated platelet activation, aggregation, and fibrin clot formation [19]. Thrombin is a key enzyme in hemostasis and has multifunctional roles in epithelial wound healing. Thrombin converts fibrinogen to fibrin, which is an integral step in fibrin clot formation. In addition to its central role in hemostasis, thrombin promotes platelet activation, aggregation, and degranulation by interacting with proteinase-activated receptors (PARs) [20,21,22].

Titanium (Ti) implants and abutments have been the “gold standard” material, but the use of ZrO_2_ as an alternative is increasing in popularity especially in patients with thin mucosal biotype [23,24]. Compared with Ti implants and abutments, ZrO_2_ implants and abutments have favorable color adaptability, physical and chemical properties, low affinity to plaque, and hypoallergenic properties [25,26,27]. Furthermore, microcirculatory dynamics in peri-implant mucosa around ZrO_2_ abutments have been shown to be comparable to those around natural teeth while blood flow is reduced around metal abutments [28]. Enhanced microcirculatory dynamics could be advantageous for the maintenance of immune function. Although ZrO_2_ is widely used as an esthetic material for implant abutment, there is limited evidence of soft tissue attachment onto the ZrO_2_ surface due to its poor adhesive potential and low reactivity [29,30]. Some studies demonstrated epithelial attachment onto the ZrO_2_ surface by additive surface modification using TiO_2_ or a glass matrix with increasing roughness [31,32]. On the other hand, studies have suggested roughness lower than 0.2 μm as a threshold to obtain a stable soft tissue seal [33,34]. Increased roughness on ZrO_2_ appeared to have enhanced osteoblast proliferation and adhesion, but epithelial cells exhibited decreased proliferation and adhesion [35,36,37,38]. To induce epithelial attachment onto a smooth ZrO_2_ surface, as in our previous study, PAR4 was a promising candidate that is now being tested in a clinical phase trial for U.S. Food and Drug Administration (FDA) approval [39]. However, due to the inertness and low reactivity of ZrO_2_, surface modification with PAR4 by conventional linking chemistries was not successful.

Electrophoretic deposition (EPD) is a traditional processing method in the ceramic industry. EPD is a two-step process [40,41,42]. In the first step, charged particles move towards the oppositely charged electrode under the effect of an externally applied electric field. In the second step, the particles deposit on the electrode. Advantages of the EPD method include a short operation time, cost-effectiveness, unneeded crosslinking agents and versatility for a variety of shapes, and the ability to coat the inner surfaces of porous structures. Our research group modified the EPD technique and employed a semi-dry transblot system to achieve Col-I coating on the titanium surface. We called our modified method “Electrophoretic fusion (EPF)” and the technique has been proven to be successful for the establishment of perpendicular collagen protrusion from the titanium nanotube surface [43,44].

In this pilot study, we employed the EPF technique to achieve immobilization of PAR4 on ZrO_2_ and to promote BL-mediated epithelial attachment. Although pure ZrO_2_ is nonconductive, dental zirconium composed of yttrium oxide (Y_2_O_3_) was expected to be conductive for the EPF. The aims of this study were to (i) verify EPF-mediated coupling of a phosphonic acid (PA) linker to ZrO_2_ and the following chemical linkage of PAR4, (ii) evaluate the activity of PAR4-funtionalized ZrO_2_ on platelet-secretions of platelet-derived growth factor—AB (PDGF-AB), and (iii) validate the epithelial attachment mode on the PAR4-ZrO_2_ surface.

## 2. Materials and Methods

### 2.1. Immobilization of the Phosphonic Acid (PA) Linker on the ZrO_2_ Surface

**Figure 1:**
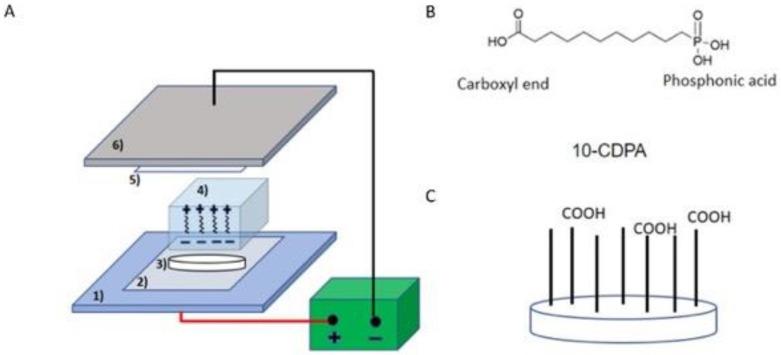
Schematic representation of PA linker Electrophoretic Fusion (EPF). (**A**) Electrophoretic Fusion set up: the CDPA linker is incorporated into PAGE gel. (**B**) Chemical structure of the CDPA linker. (**C**) Immobilization of the phosphonic acid end of CDPA onto the ZrO_2_ surface by EPF leaving the free carboxyl end.

Round ZrO_2_ discs that were 10 mm in diameter and 0.39 mm in thickness (Cusp Dental, Malden, MA, USA) were prepared for qualitative and quantitative determination of platelet aggregation and epithelial cell attachment in a 24-well plate format. The ZrO_2_ discs were sequentially cleaned ultrasonically in 0.5% sodium dodecyl sulfate (SDS; Sigma, St. Louis, MO, USA), deionized water, acetone (Sigma, St. Louis, MO, USA), and 100% ethanol (Sigma, St. Louis, MO, USA) for 20 min in each solvent. A part of the samples was prepared by conventional passive soaking for self-assembled monolayers (SAMs). Cleaned ZrO_2_ discs were incubated for 3 h in 1 mM PA linker solution (CDPA, Dojindo Molecular Technology, Rockville, MD, USA) with gentle rocking at 70 °C. The ZrO_2_ discs were washed in ethanol 4 times, air-dried, and heated at 120 °C for 24 h in a drying oven. For other test samples, an electrophoretic fusion (EPF) method was applied to immobilize CDPA onto the ZrO_2_ surface (Figure 1). Then, 1 mM of CDPA was incorporated into 6% Bis Tris gel with neutral pH during fabrication to endow negative charges to the phosphonic acid end and to keep the carboxyl acid group neutral. The transfer unit was assembled (from the bottom) in the following order: (1) anode of a semi dry blotter (Trans-Blot Turbo Transfer System^®^, BIO-RAD Laboratory, Hercules, CA, USA); (2) 1 × Tris glycine buffer (TGB)-wetted filter paper; (3) ZrO_2_ discs; (4) 6% Bis Tris gel (BIO RAD Laboratory, Hercules, CA, USA) with 1 mM CDPA; (5) TGB-wetted filter; and (6) cathode. Electroblotting transfer of CDPA was carried out under a constant voltage at 25 V for 2 min. After the transfer, the specimens were washed 3 times with PBS.

### 2.2. Evaluation and Analyses of CDPA-Fused ZrO_2_

CDPA at the ZrO_2_ surface was analyzed by Fourier Transform Infrared Spectroscopy in Attenuated Total Reflection mode (FTIR-ATR) (Lumos FTIR Microscope, Bruker, MA, USA). The ATR crystal was a single bounce germanium crystal with a 125-micron diameter. CDPA-fused ZrO_2_ discs were examined under FTIR with a 4 cm^−1^ resolution. Sixteen scans were averaged together for each data point over the range of 600–4000 cm^−1^.

According to the FTIR peak database, the presence of CDPA on ZrO_2_ discs was determined by vibrations of chemical compounds from carboxylic acid in CDPA (C=O stretch vibration absorption peak from 1760–1690 cm^−1^, C-O stretch vibration absorption peak from 1320–1210 cm^−1^, O-H bend in the region of 1440–1395 cm^−1^).

The test sample data were processed by subtracting the FTIR spectra of the control ZrO_2_ sample. After baseline correction, the peaks from carboxylic acid of CDPA were analyzed using OPUS software (Bruker, Billerica, MA, USA).

### 2.3. Coupling of the PA Linker and PAR4

The carboxyl residues in CDPA were activated by rotating the ZrO_2_ discs at room temperature for 2 h in 0.2 M N-hydroxysuccinimide (NHS; Sigma, St. Louis, MO, USA) and 0.25 M 1-ethyl-3-(3-dimethylaminopropyl) carbodiimide (EDC; Sigma, St. Louis, MO, USA) dissolved in dimethylformamide (DMF; Sigma, St. Louis, MO, USA). After washing 4 times in DMF, activated CDPA-fused ZrO_2_ samples were incubated with 0.1 mM PAR4 (AYPGKF-NH_2_; Sigma, St. Louis, MO, USA) in DMF solution with gentle rotation at room temperature for 70 min. Then, the samples were washed 4 times with DMF, air-dried, and stored at 4 °C.

### 2.4. Evaluation and Analysis of PAR4-Fused ZrO_2_

PAR4-fused ZrO_2_ samples were evaluated and analyzed by FTIR-ATR. The presence of both CDPA and PAR4 was determined by carboxylic acid from CDPA (C=O stretch vibration absorption peak from 1760–1690 cm^−1^, C-O stretch vibration absorption peak from 1320–1210 cm^−1^, O-H bend in the region of 1440–1395 cm^−1^ and 3300–2500 cm^−1^) and amide II bond from PAR4 (N-H stretching vibration absorption peak from 3700–3500 cm^−1^). The test sample data were processed by subtracting the FTIR spectra of the intact bare ZrO_2_ disc sample. After baseline correction, the peaks from the carboxylic acid of CDPA and amide II of PAR4 were calculated using OPUS software.

### 2.5. Preparation of PRP

Human whole blood with 10% anticoagulant citrate dextrose (ACD) was purchased from BioIVT (ELEVATING SCIENCE^®^, Westbury, NY, USA). Each whole blood sample was collected from a single donor without any identification involved. Platelet-rich plasma (PRP) was prepared according to a previously published protocol. (51) With the use of a centrifuge machine (Salvin Dental, Charlotte, NC, USA), tubes containing whole blood were centrifuged at 3600 rpm, which generated 1000 g-force for 2.5 min and 15 s at room temperature. After separation of the whole blood into two layers, the light-yellow supernatant plasma layer was transferred to new tubes with no anti-coagulant. A second centrifugation was done at the same setting for another 5 min. Afterwards, the upper layer of platelet-poor plasma (PPP) was collected, leaving red pellets containing concentrated platelets at the bottom. Inactivated PRP was prepared by resuspension of pellets with 6 mL of PPP. Prior to use, CaCl_2_ in PBS was added for a final concentration of 14.3 mg/mL to counteract the 10% ACD to produce activated PRP.

### 2.6. Platelet Adhesion and Activation on ZrO_2_ discs

Control and PAr4-ZrO_2_ discs were placed in a 24-well plate (*n* = 5), and 1 mL of PRP was inoculated onto the samples. The plate was placed on a rocking table at 37 °C for 60 min. At the end of the incubation, unbound cells were washed off with PBS and plasma was collected for evaluation of platelet-derived growth factor-AB (PDGF-AB) with sandwiched enzyme-linked immunosorbent assay (ELISA) kits (R & D Systems, Minneapolis, MN, USA). The platelets that adhered on the ZrO_2_ surface were fixed in 4% paraformaldehyde (PFA), washed in water, and dehydrated in an ethanol series to 100% (75%, 80%, 85%, 90%, 95%, and 100%, 20 min in each incubation) for scanning electron microscopy (SEM, Zeiss Supra 55VP field emission scanning electron microscope; ZEISS, Oberkochen, Germany).

### 2.7. Human Gingival Epithelial Cell Adhesion to the ZrO_2_ Surface

Cells of the human gingival epithelial cell line OBA9 (a gift from Prof. Murakami at Osaka University School of Dentistry, Japan) [45] were maintained in keratinocyte-SFM medium (Thermo Fisher Scientific, Waltham, MA, USA) at 37 °C in 5% CO_2_ and 95% atmospheric air. OBA9 cells, passaged between 4 and 10 times, were used in this study. OBA9 cells were cultured on ZrO_2_ discs with and without the surface modification (*n* = 7 for each group) on which PRP was previously incubated for 60 min in a 24-well format. Unbound platelets were washed off with PBS from the ZrO_2_ discs before OBA9 cells were inoculated. OBA9 cells were then cultured on the platelet-doped ZrO_2_ discs for 48 h. At 48 h, unbound cells were washed off with PBS. Two specimens from each group were fixed with 4% PFA for SEM analysis. (Zeiss Supra 55VP; Zeiss, White Plains, NY, USA). Dehydration with ethanol was not performed on other OBA9 specimens (*n* = 2 per group) to avoid nonspecific staining in immunocytochemistry. The basement membrane (BM) and tight junction proteins of laminin-5 (Ln5) were stained with fluorescence-labeled antibodies (laminin 5-antibody, DyLight 488 conjugate: Thermo Fisher Scientific, Waltham, MA, USA). The cell nuclei were counter-stained with DAPI (FluoroshieldTM with DAPI; Sigma, St. Louis, MO, USA) and imaged under a confocal microscope. The number of attached cells on the rest of the samples (*n* = 3 per group) was calculated using a cell luminescent viability kit (CellTiter-Glo^®^, Promega, Madison, WI, USA).

### 2.8. Statistical Analyses

To compare the significant differences in the number of cells attached to the specimen surfaces and the extent of PDGF-AB release, a two-tailed test (*t*-test) was conducted. The significance level adopted was 5% for all tests.

## 3. Results

### 3.1. Immobilization of CDPA on the ZrO_2_ Surface

**Figure 2:**
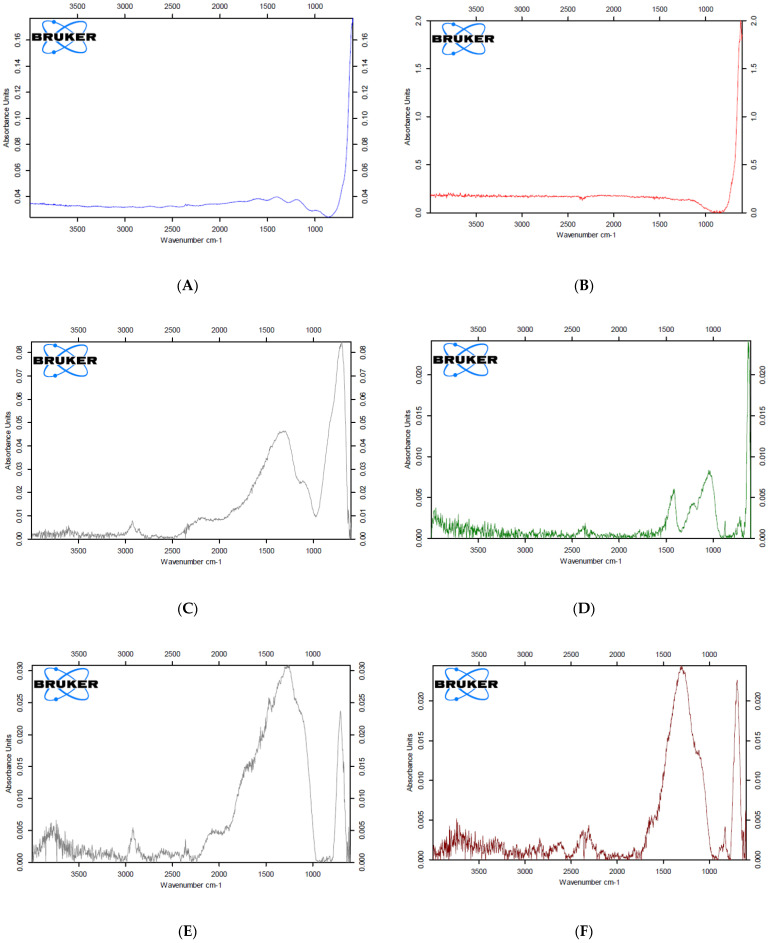

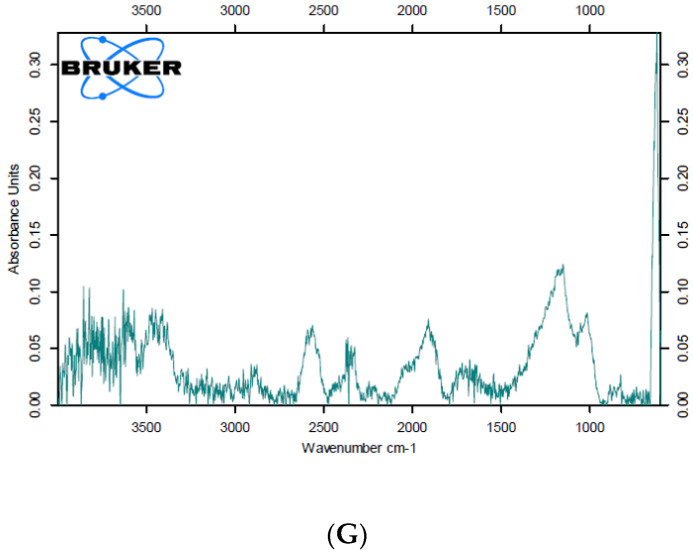
FTIR spectra of ZrO_2_ surfaces with/without the CDPA linker and PAR4. (**A**) Non-treated ZrO_2_ (Control). (**B**) CDPA-doped ZrO_2_ by self-assembled monolayers (SAMs). (**C**) CDPA-fused ZrO_2_ by EPF (Test sample 1). (**D**) CDPA-fused ZrO_2_ by EPF (Test sample 2). (**E**) CDPA-fused ZrO_2_ by EPF + PAR4 (test sample 1). (**F**) CDPA-fused ZrO_2_ by EPF + PAR4 (test sample 2). (**G**) CDPA-fused ZrO_2_ by EPF + PAR4 (test sample 3).

CDPA-fused ZrO_2_ and non-fused control samples were analyzed by FTIR-ATR. Carboxylic acid from CDPA showed strong peaks corresponding to O-H stretching, carbonyl stretching of C=O, and C-O stretching based on FTIR. The nontreated control and SAM test sample did not show any O-H, C=O or C-O peaks, only a Zr peak at around 420 cm^−1^ (Figure 2A,B). EPF test sample 1 revealed a Zr peak at around 420 cm^−1^ as well as one broad peak ranging from 1100 cm^−1^ to 2300 cm^−1^ which corresponded to the combination of carboxylic end peaks from C-O bonds, O-H bonds, and C=O bonds (Figure 2C). EPF test sample 2 also showed two peaks ranging from 1100 cm^−1^ to 1600 cm^−1^, which corresponded to C-O bonds, O-H bends, and C=O bonds, as well as a Zr peak at around at around 420 cm^−1^ (Figure 2D). Two test samples subjected to the EPF method indicated C-O bonds, O-H bends, and C=O bonds from CDPA while the intact bare and SAM test sample showed a Zr peak only. The presence of CDPA was confirmed by FTIR, and therefore, the CDPA coating on ZrO_2_ via EPF was verified. 

CDPA-PAR4 fused test samples were analyzed by FTIR. PAR4 (AYPGKF-NH_2_) is characterized by amide II bonds in FTIR analysis. Test sample 1 had an amide peak at around 3700 cm^−1^ and a strong wide bend ranged from 1000 to 2500 cm^−1^ that corresponded to combination of peaks from carboxylic acid in CDPA and amino acid side chains of PAR4 (Figure 2E). Test sample 2 showed an amide peak at around 3700 cm^−1^ and a strong wide bend that ranged from 1000 cm^−1^ to 1750 cm^−1^ (Figure 2F). Test sample 3 showed an amide peak at around 3600 cm^−1^ and peaks at 1200 cm^−1^ and 1700 cm^−1^ (Figure 2G). All three test samples had amide peaks from 3700–3500 cm^−1^, which verified the presence of PAR4. The successful coupling of PAR4 with CDPA on the ZrO_2_ surface was confirmed by FTIR.

### 3.2. Platelet Activation and Aggregation on the ZrO_2_ Surface

**Figure 3:**
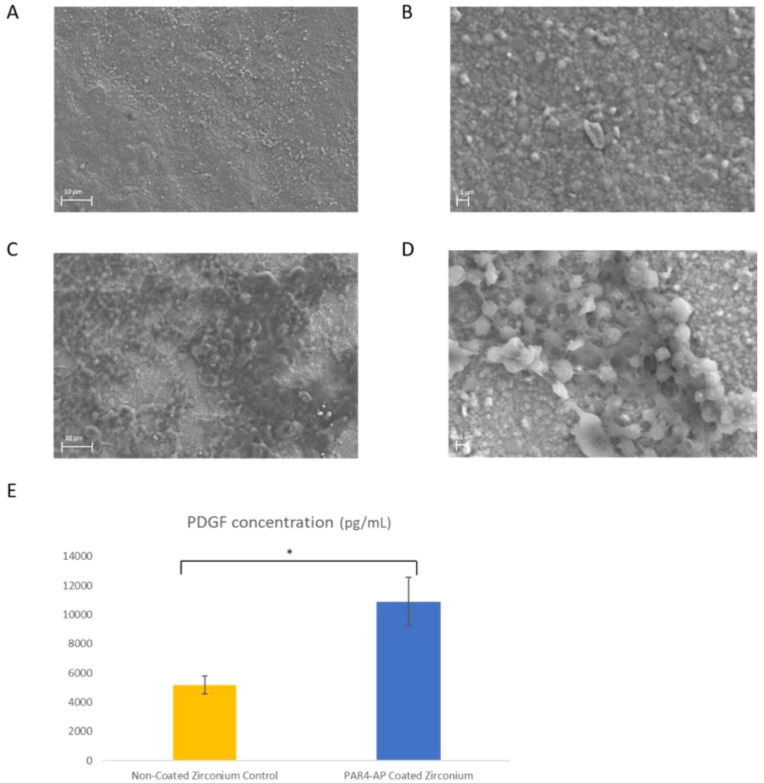
Platelet aggregation and activation on ZrO_2_. (**A**) SEM image: Control ZrO_2_, magnification ×3.00 K. (**B**) SEM image: Control ZrO_2_, magnification ×10.00 K. (**C**) SEM image: PAR4-fused ZrO_2_, magnification ×3.00 K. (**D**) SEM image: PAR4-fused ZrO_2_, magnification ×10.00 K. (**E**) ELISA results of the PDGF-AB concentration (pg/mL): Pure ZrO_2_ (yellow) and PAR4-fused ZrO_2_ (blue) samples were incubated with PRP for 60 min. Data represent the mean ± SD. * *p* < 0.05.

Platelet aggregation and activation on the ZrO_2_ surface were identified by SEM and ELISA after 60 min PRP incubation. On the non-modified ZrO_2_ surface, no form of platelet aggregation was observed by SEM (Figure 3A,B). On the other hand, platelet aggregation was observed on the PAR4-fused ZrO_2_ surface. Dense aggregation of platelets around 1–2 μm in diameter was clearly observed by SEM (Figure 3C,D). A significantly higher amount of PDGF-AB was released from PAR4-fused ZrO_2_ compared to control ZrO_2_ after 60 min incubation (*p* = 0.001) (Figure 3E).

### 3.3. Epithelial Attachment to the ZrO_2_ Surface

**Figure 4:**
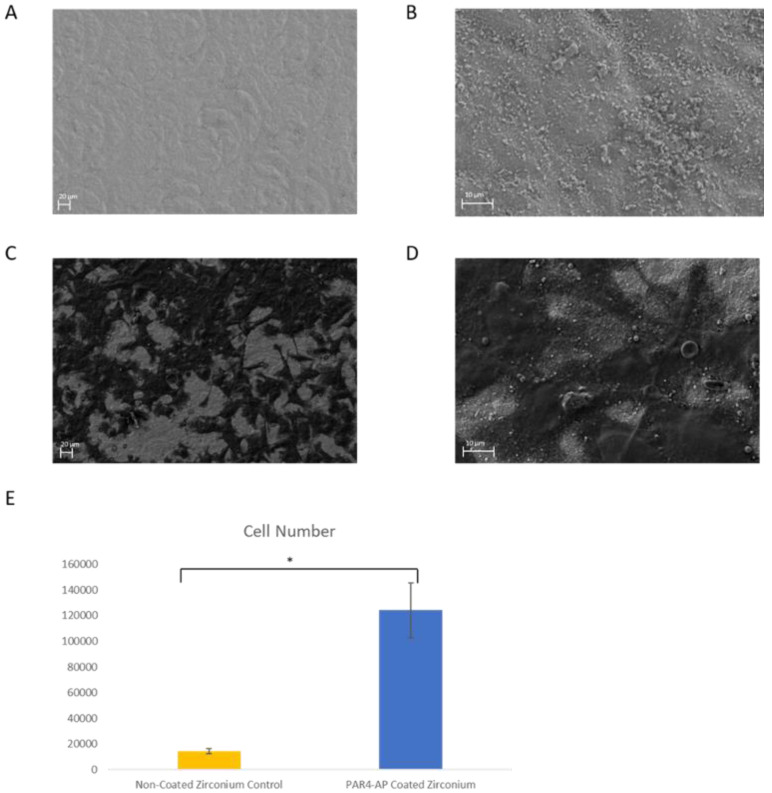
OBA9 epithelial cell attachment on ZrO_2_. (**A**) SEM image: Control ZrO_2_, magnification ×500. (**B**) SEM image: Control ZrO_2_, magnification ×3.00 K. (**C**) SEM image: PAR4-fused ZrO_2_, magnification ×500. (**D**) SEM image: PAR4-fused ZrO_2_, magnification ×3.00 K. (**E**) Number of attached OBA9 epithelial cells: Pure ZrO_2_ (yellow) and PAR4-fused ZrO_2_ (blue) samples were incubated with PRP for 60 min. Subsequently, OBA9 cells were inoculated and cultured for 48 h. Data represent the mean ± SD of pure ZrO_2_ and PAR4-fused ZrO_2_. * *p* < 0.05.

**Figure 5:**
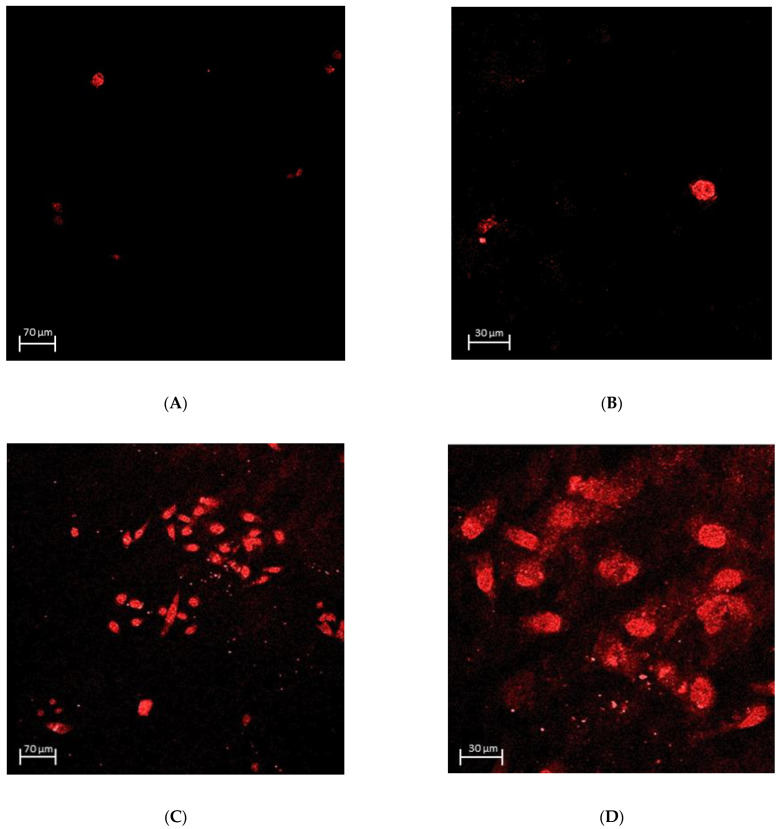
Immunocytochemical staining of Bm-Ln5 on the Zr surface incubated with OBA9 cells for 48 h. (**A**) Control ZrO_2_: no clusters of epithelial cells. (**B**) Control ZrO_2_: round shape of epithelial cells. (**C**) PAR4-fused ZrO_2_: clusters of epithelial cells observed. (**D**) PAR4-fused ZrO_2_: prominent elongated shape of epithelial cells.

**Figure 6:**
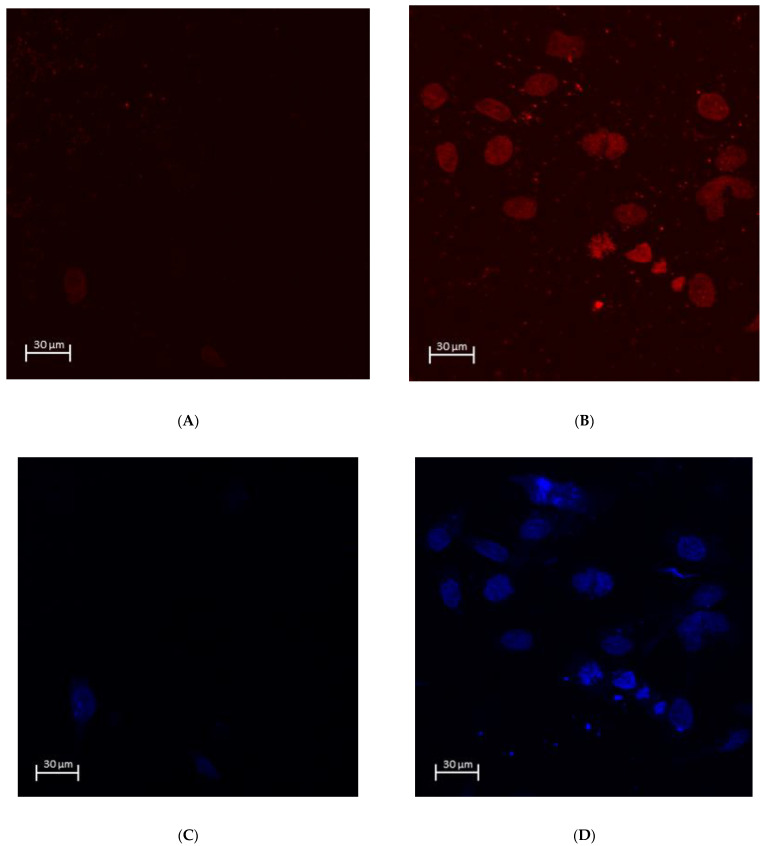

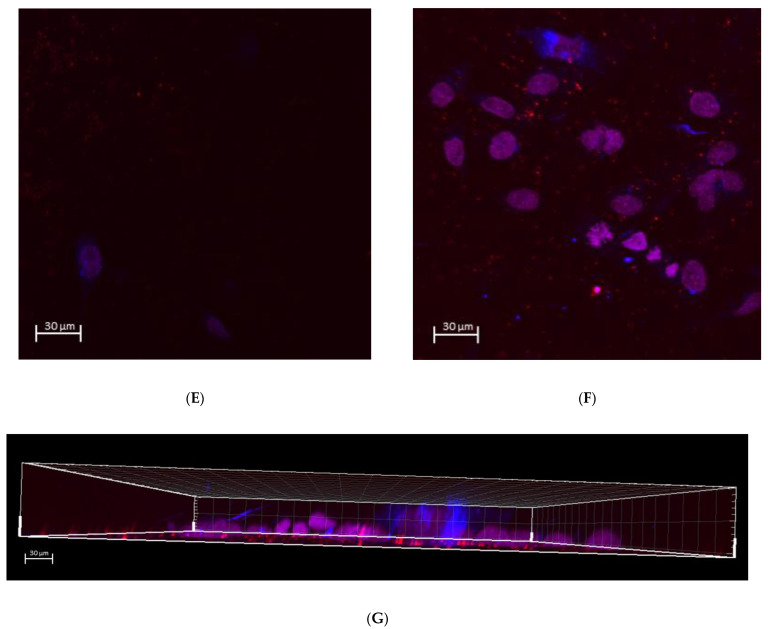
Immunocytochemical staining of Bm-Ln5 with DAPI on the Zr surface incubated with OBA9 cells for 48 h. (**A**) Control ZrO_2_ (Ln5): no clusters of epithelial cells. (**B**) PAR4-fused ZrO_2_ (Ln5): prominent elongated shape of epithelial cells. (**C**) Control ZrO_2_ (DAPI): no clusters of epithelial cells. (**D**) PAR4-fused ZrO_2_ (DAPI): prominent elongated shape of epithelial cells. (**E**) Control ZrO_2_ (Ln5/DAPI merge): no clusters of epithelial cells. (**F**) PAR4-fused ZrO_2_ (Ln5/DAPI merged): prominent elongated shape of epithelial cells. (**G**) PAR4-fused ZrO_2_ cross-sectional image along the Z axis of the immunocytochemical staining of BM-Ln5/DAPI: BL-Ln5 (red) adhesion to the surface observed while counter-stained nuclei with DAPI (blue) were observed on top of the BL-Ln5 layer (red). This indicated BL-mediated attachment to the ZrO_2_ surface.

The number of attached OBA9 epithelial cells was significantly greater on the PAR4-fused ZrO_2_ surface compared to that on the control (*p* = 0.002) (Figure 4E). The mode of OBA9 epithelial cell attachment on the ZrO_2_ surface was evaluated by SEM and immunocytochemistry. No epithelial cell attachment was observed on the control ZrO_2_ surface by SEM (Figure 4A,B). In addition, the BL-component of laminin-5 was not detected on control ZrO_2_ by confocal microscopy (Figure 5A,B). On the other hand, widely spread dense colonies with elongated epithelial cell-like morphology were seen on the PAR4-fused ZrO_2_ surface (Figure 4C,D). Each epithelial cell with flat and elongated morphology was 12–17 μm diameter. Confocal microscopy captured clusters of epithelial cells on the PAR4-fused ZrO_2_ surface (Figure 5C,D). In addition, epithelial BL attachment on the PAR4-surface was identified with BL-Ln5 (Figure 6B,D,E). BL-Ln5 adhesion to the surface was observed on the PAR4-fused ZrO_2_ surface while counter-stained nuclei with DAPI were found on top of the BL-Ln5 layer (Figure 6G).

## 4. Discussion

In this study, we applied CDPA as a crosslinker between the ZrO_2_ surface and PAR4. CDPA is an alkyl phosphonic derivative containing carboxyl acid at the contralateral end. Since the presence of polar sites on carboxyl acid can compete for the ZrO_2_ surface, preventing well-organized monolayers [46], the pH of PAGE was set at neutral where divalent phosphonic anions and the monovalent carboxyl end predominated in EPF. At the phosphonic acid end, there were three oxygen atoms in the phosphonic acid moiety (one phosphoryl oxygen and two hydroxyl oxygen atoms), and therefore up to three oxygen atoms can bind to the ZrO_2_ surface. In other words, phosphonic acids can bind to the ZrO_2_ surface in a monodentate, bidentate or tridentate mode [47]. Under the neutral pH of PAGE in EPF, divalent phosphonic anions may be deprived of electrons from the third -OH on the anode; thus, tridentate binding may occur. We hypothesized that the differences in charges in EPF promoted the formation of high-coverage phosphorus directed by the CDPA monolayers on the ZrO_2_ surface.

CDPA is a self-assembled monolayer (SAM) forming a chemical linker that has phosphonic acid and carboxylic acid termini. Robust assembly spontaneously occurs at the phosphonic acid terminus on various metal oxides including TiO_2_, ZnO_2_, and Al_2_O_3_. CDPA is supposed to be compatible with ZrO_2_ as claimed by the manufacturer. However, a small peak of CDPA was detected by FTIR when the ZrO_2_ disc was immersed in 1 mM of the ethanol solution as indicated in the manufacturer’s protocol. The failure might suggest an antifouling/antimicrobial feature of ZrO_2_ due to the chemically inert surface. Then, we tested the EPF method that introduces a phosphonic acid residue onto the ZrO_2_ anode. During polyacrylamide gel (PAGE) fabrication, CDPA was suspended into the PAGE where phosphonic acid derivatives were negatively charged in divalent form. Under the neutral pH of the electrophoresis running buffer, phosphonic acid residues dissociated into divalent anions, while carboxylic acid dissociated into monovalent anions. Therefore, the divalent anion residue should move towards the positive ZrO_2_ anode against the flow resistance of the PAGE gel and enhance the reactivity. Thus, phosphonic acid-directed immobilization should leave the carboxylic terminus free to couple with the PAR4 peptide. The principle of EPF through PAGE was so simple and classical yet allowed immobilization of CDPA followed by successful amide coupling of the PAR4 peptide without modification of the surface roughness.

Under SEM, aggregation of platelets was not observed on the non-treated smooth ZrO_2_ surface (Figure 3A,B). This was expected as zirconia is known to be inert and has low reactivity. The success of epithelial attachment onto the ZrO_2_ surface lies in the attachment and aggregation of platelets, which are the critical steps for fibrin clot formation and ultimately epithelial attachment. After successful deposition of CDPA and PAR4 on the ZrO_2_ surface, platelet attachment and aggregation were observed under SEM (Figure 3C,D). In addition, PAR4-activated platelet attachment and aggregation were confirmed by the burst of PDGF release (Figure 3E).

As we expected, epithelial cells attached on the platelet-aggregated ZrO_2_ surface via BL (Figure 6B,D,F,G). The morphology and clustering of OBA9 cells indicated the adhesion of mature epithelial cells on the ZrO_2_ surface (Figure 4C,D and Figure 6B,D,F,G).

The limitations of this study are as follows: first, all experiments were performed in an in vitro setting, which may not provide an accurate reflection of in vivo settings. Second, the stability of the established epithelial sealing on ZrO_2_ should be further studied with comparison to Ti and natural teeth in future experiments. The significance of this pilot study is the verification of the CDPA-linked immobilization of the PAR4 peptide to ZrO_2_, as well as the biological effects on BL-mediated epithelial attachment via platelet aggregation and activation on the ZrO_2_ surface in situ. A future study will further investigate the maintenance of gingival attachment on ZrO_2_-abutment/crowns at an esthetic level.

## 5. Conclusions

In this study, successful phosphonic acid residue-directed immobilization of the CDPA linker on the ZrO_2_ surface was achieved via the EPF method, which allowed the subsequent coupling of the PAR4 peptide. The resultant surface was bioactive, enabling effective platelet aggregation, activation, and robust epithelial basal lamina attachment. The significance of our study is the ability of the EPF method to convert the inert ZrO_2_ surface to a bioactive surface, inducing epithelial cell attachment. In addition, the versatility of the EPF method enables any linear compound with electric polarity to be used as a chemical linker for surface modification.

## Data Availability

Not applicable.
